# Detectable viral load associated with unmet mental health and substance use needs among trans women living with HIV in San Francisco, California

**DOI:** 10.1186/s12905-024-02885-8

**Published:** 2024-01-22

**Authors:** Erin C. Wilson, Glenda N. Baguso, Jerry Quintana, Bow Suprasert, Sean Arayasirikul

**Affiliations:** 1 Trans Research Unit for Equity, Center for Public Health Research, San Francisco Department of Public Health, San Francisco, USA; 2 Department of Epidemiology & Biostatistics, University of California, San Francisco, USA; 3 Department of Health, Society, and Behavior, Program in Public Health, University of California, Irvine, USA

**Keywords:** Transgender women, HIV, Mental health, Substance use

## Abstract

**Background:**

Substance use and mental distress are known barriers to HIV care engagement among trans women. Less is known about access and utilization of mental health and substance use care among trans women and the relationship between unmet behavioral health needs and HIV viral suppression. We examined the relationship between mental health and substance use on HIV viral load among trans women living with HIV. We also examined the relationship between mental health and substance use services needs with HIV care engagement and having a detectable viral load by comparing engagement in care cascades.

**Methods:**

Data are from a 2022 baseline assessment for an intervention with trans women living with HIV (*n* = 42) in San Francisco. Chi-Squared or Fisher’s exact tests were conducted to determine associations between HIV viral load, mental health, and substance use. We also examine characteristics associated with each step in the HIV, mental health, and substance use care cascades.

**Results:**

Most participants were trans women of color (85.7%), 40 years of age or older (80.9%), with low income (88.1%), and almost half were unstably housed (47.6%). Of the 32 participants who screened positive for depression, anxiety and/or psychological distress, 56.3% were referred for mental health services in the past 12 months. Of those who were referred, 44.4% received mental health services. Of the 26 participants who screened positive for a substance use disorder, 34.6% were referred to substance use services in the past 12 months. Of those referred, 33.3% received substance use services in the past 3 months. Latina trans women had a low referral rate to meet their mental health needs (50%) and only 16.7% of African American/Black trans women who screened positive for a substance use disorder were referred for services, while trans women of other race/ethnicities had high referral and services utilization. No significant results were found between HIV viral load and screening positive for a mental health disorder. Methamphetamine use was statistically associated with having a detectable HIV viral load (*p* = 0.049).

**Conclusions:**

We identified significant unmet mental health and substance use services needs and noted racial/ethnic disparities in the context of high HIV care engagement among trans women living with HIV. We also found that methamphetamine use was a barrier to having an undetectable viral load for trans women living with HIV. To finally end the HIV epidemic, integration of behavioral health screening, linkage, and support are needed in HIV care services for populations most impacted by HIV, especially trans women.

**Trial registration:**

NCT, NCT 21–34,978. Registered January 19, 2022.

## Background

Transgender (trans) women are highly impacted by HIV, with a 2019–2020 HIV prevalence of 42% among trans women across 7 cities in the U.S. [1]. Only 67.2% of trans people in the U.S. were virally suppressed at their most recent viral load test [2]. Despite San Francisco offering a range of important health services like HIV specialty and gender-affirming care to trans women with and without health insurance, almost a quarter of trans women in our city remain virally detectable as of 2020 [3], and many suffer from threats to wellness due to stigma and discrimination. Almost half of trans women in 2020 were recently experiencing homelessness (47%), extremely low income (42.4%), and more than half (64.3%) recently injected drugs based on the latest National HIV Behavioral Surveillance data for trans women [3].

Stigma and discrimination often results in substance use and poor mental health. Psychological distress among trans women is directly related to structural barriers from anti-trans discrimination leading to housing, food, and income insecurity [4, 5]. Data from the National Transgender Discrimination Survey show that trans people who face a high level of stigma and discrimination are more likely to suffer psychological distress and engage in substance use [6]. A recent study in San Francisco found that having a mental health condition was significantly associated with stimulant use over time among trans women [7].

Problems with mental health and substance use are also associated with poor HIV care outcomes in other populations, but less is known about this connection for trans women and the effects on HIV care engagement. Research finds that mental health is an important predictor of viral suppression [8, 9]. Data also show that more trans people living with HIV experienced symptoms of depression and anxiety than people of any other gender group across the U.S. [10]. Yet no study that we know of examines the relationship between mental health and HIV outcomes among trans women living with HIV through the lens of the HIV care cascade. Although research with sexual and other minoritized communities exists on the effect of substance use on HIV care outcomes, little research has been exclusively conducted with trans women [11, 12]. Among studies of trans women living with HIV, substance use is prevalent [13]. One recent study found that stimulant use was associated with linkage to HIV care delays and any drug use was associated with fewer HIV care visits among trans women living with HIV [14]. More research is needed to examine associations between mental health and substance use services needs for trans women living with HIV.

This study was conducted to fill important gaps in what we know about the relationship between mental health, substance use, and HIV care outcomes among trans women living with HIV. We examined associations between substance use, mental health, and HIV viral load to determine if mental health distress and substance use were associated with a detectable HIV viral load. We also compared HIV care engagement to mental health and substance use referrals and treatment engagement to examine unmet need. Lastly, we explored socio-demographic factors associated with the unmet need for mental health, substance use referrals, and treatment services among trans women living with HIV.

## Methods

Data for this study were gathered from the baseline assessment of a pilot intervention to improve mental health and substance use services and treatment engagement among trans women living with HIV conducted between January and April 2022. Trans women were recruited through referrals from gender-affirming HIV care public health clinics in San Francisco. Trans women were screened prior to the baseline survey. The eligibility screener included demographics, self-reported HIV visit attendance in the past 12 months, current viral load, the PHQ-4 screener for anxiety and depression, and questions indicating whether participants drank or used substances more than they meant to in the past year or had the desire to cut down drinking or substance use in the past year. Eligible participants a) identified as a trans woman, b) were 18 years or older, c) were living with HIV, d) reported that they used more alcohol or substance use than they meant to in the past year or expressed a desire to reduce their drinking or substance use behaviors, or had an PHQ-4 score indicating anxiety or depression, d) received healthcare at a public health clinic, and e) was either not linked to HIV care, missed an HIV care appointment in the past year, or was not currently on antiretroviral therapy (ART) or had a detectable viral load. After excluding participants who did not meet the eligibility criteria, the total final sample size for the analyses was 42. The incentive for the baseline intervention visit, including collection of survey data, was $100.

### Measures

An interviewer-administered survey was conducted to capture basic demographics (e.g., age, race/ethnicity, employment, and living situation) and other social determinants of health (e.g., experience of food insecurity, current sex work). Food insecurity was asked as “In the past 12 months, did you ever cut the size of your meals or skip meals because there wasn’t enough money for food?”. Sex work was defined as true when participants indicated they received income from sex work. The baseline survey also assessed access to HIV, mental health, and substance use treatment among trans women in San Francisco. In addition to self-report measures, secondary data from the electronic medical records were used to determine a) indicators of HIV care including number of HIV clinic visits, number of no-shows for HIV care visits, viral load, CD4 count, and b) mental health or substance use diagnoses, and treatment referrals and utilization.

#### Substance use

The 3-item AUDIT Alcohol Consumption Questions (AUDIT-C) [15] was used to screen for alcohol use disorder (AUD). Total score was created with a range from 0 to 12. A cut-off score of 3 or greater was used to indicate alcohol use disorder among transgender women [16, 17]. To measure other substances used, we asked “Which recreational substances have you used in the past year?”. Response options were (1) Methamphetamines (speed, crystal), (2) Cocaine, (3) Cannabis (marijuana, pot), (4) Opioids (heroin, oxycodone, methadone, etc.), (5) Inhalants (paint thinner, aerosol, glue), (6) Hallucinogens (LSD, mushrooms), (7) Tranquilizers (valium), and (8) Other. Participants were allowed to select more than one type of substance. A new variable, polysubstance use, was created if multiple recreational substances were selected from the above list, excluding cannabis. The 10-item Drug Abuse Screening Test (DAST-10) was used to screen for a drug use disorder. Answer choice “yes” was assigned a value of 1 and answer choice “no” was assigned a value of 0. Traditionally, question 3 of DAST-10 asked, “Are you always able to stop using drugs when you want to?” and a reverse score was assigned. In our current study, we reverse the question instead of the score. Our question 3 asked, “Are you unable to stop using drugs when you want to?”. The sum score was created and ranged from 0 to 10. A cut-off score of 6 or greater was used identify a drug use disorder [18].

#### Mental health

The 4-item patient health questionnaire for anxiety and depression (PHQ-4) was used to screen for depression and anxiety. A total score with a possible range from 0 to 12 was created. Scores of 0 to 2 were rated as not clinically significant for anxiety or depression symptom level, 3 to 5 were rated as mild symptom level, 6 to 8 were rated as moderate symptom level, and 9 to 12 were rated as severe symptom level. PHQ-4 can also be assessed separately. A sum score of the first two questions was created to detect presence of an anxiety disorder and a sum score of the last two questions was created to detect a depressive disorder. A cut-off of 3 was used to determine whether any anxiety and depressive disorder were detectable [19–21]. In addition, the 6-item Kessler Psychological Distress Scale (Kessler) was used to measure psychological distress based on how often participants felt 6 emotional states over the past 30 days. A total score with a possible range between 0 and 24 was created. A score of 0 indicated no psychological distress, whereas scores of 1 to 5 indicated low psychological distress, 6 to 10 indicated moderate psychological distress, and 11 to 24 indicated high psychological distress [22]. A cut-off score of 13 or greater was also used to indicate serious psychological distress [23]. History of mental and/or substance use diagnosis was assessed using responses to the question “Have you been diagnosed with any of the following conditions by a health professional (e.g., primary care doctor, psychiatrist, psychologist, etc.)?”

#### HIV viral load

HIV viral load data were abstracted from the medical record at baseline for each participant. HIV viral load was categorized into [1] unknown if the most recent HIV viral load test was conducted over 180 days prior to their study initiation date, [2] detectable if viral load was ≥50 copies/mL, or [3] undetectable if there was no virus detected. Most people can lower their viral load to undetectable between 1 to 6 months of initiating ART, although this depends on the individual. The most recent HIV report from San Francisco found that 75% of newly diagnosed people reached an undetectable HIV viral load status within 6 months [24]. Furthermore, many people with an undetectable HIV viral load can become detectable within weeks of stopping HIV treatment.

#### Cascades

The HIV, mental health, and substance use care cascades were developed to identify gaps in care for trans women. The cascades start off with the total number of trans women who ever had a [1] HIV diagnosis, [2] screened positive for a mental health disorder, and [3] screened positive for a substance use disorder (including an alcohol or drug use disorder). Screening positive for a mental health disorder was defined as having moderate to high anxiety or depression (PHQ-4 score ≥ 6) or having high psychological distress (Kessler score ≥ 11) or having PTSD (PTSD score ≥ 4). Screening positive for a drug use disorder was defined as using drugs daily or almost daily or unable to stop using substances or having an AUDIT-C score ≥ 3. To determine whether participants were referred for mental health or substance use treatment, we asked “In the last 12 months, has anyone at your HIV care clinic referred you to mental health services (e.g., counseling, treatment, support groups for depression or other issues)?”. For substance use services, the question was replaced with substance use services (e.g., counseling, treatment). Currently receiving care was defined as those who reported receiving HIV care, mental health counseling, or substance use treatment in the past 3 months.

### Analysis

Using STATA version 17.0 (College Station, TX), we provide descriptive statistics of key measures including baseline demographic characteristics, mental health, and substance use scores and diagnoses. Chi-squared or Fisher’s exact tests were conducted to assess differences in mental health and substance use between for those with an undetectable viral load compared to those with an unknown or detectable HIV viral load.

We further conducted bivariate analyses using Chi-squared or Fisher’s exact tests to determine differences in the proportion of key characteristics (e.g., experience of food insecurity, engagement in sex work) for each step in mental health and substance use treatment care cascades. A significant level was set at alpha = 0.05.

The protocol was reviewed and approved by the Institutional Review Board of University of California, San Francisco (protocol # 18–26,447). Written consent was obtained along with a signed HIPAA authorization for electronic medical records abstraction.

## Results

Table 1 depicts baseline characteristics of trans women in our study. About one-third (31.0%) identified as Black/African American, followed by 28.6% Latina, 14.3% Asian/Pacific Islander (PI), and 14.3% White. Almost one-third (33.3%) had less than a high school degree, 31.0% had a high school degree, and 35.7% had some college, associate degree, or higher education. Most trans women were not employed (88.1%). All participants were ages 30 years or older with a third falling in the 50–59 years old age group (33.3%). About half of participants were stably housed and rented or owned (52.4%) and half lived in a shelter/motel, were homeless, or living on the street (47.6%). Half of participants earned equal to or less than $1000 a month, and only 11.9% made more than $2,000 a month. More than half of participants reported food insecurity experience in the past 12 months (59.5%). More than half of participants said that their main source of income was from benefits such as unemployment, disability, and retirement (78.6%). About a quarter of participants said that their main source of income was from a partner, ex-partner, family, or friend (23.8%). About one-fifth of participants said sex work was their main source of income (19.1%). About three-fourths of participants had Healthy San Francisco (78.6%), a program that provides health coverage to uninsured San Francisco residents. About half of participants had Medi-Cal (47.6%) and/or Medicare public health insurance coverage (59.5%).

**Table 1:** Baseline characteristics of trans women living with HIV in San Francisco, mSN study, 2022, *N* = 42

Characteristics	Trans women *N* = 42 (%)
Race/Ethnicity	
African American/Black	13 (31.0)
Latina	12 (28.6)
White	6 (14.3)
Asian/Pacific Islander	6 (14.3)
Native American	1 (2.4)
Multiracial/ethnic	4 (9.5)
Education	
< High school diploma	14 (33.3)
High School diploma or GED	13 (31.0)
> High School diploma	15 (35.7)
Employment	
Not currently employed in formal sector	37 (88.1)
Currently employed	5 (11.9)
Age	
Average	50.31 (10.7)
Median	50.5
Age group	
30–39	8 (19.1)
40–49	11 (26.2)
50–59	14 (33.3)
60+	9 (21.4)
Living situation	
Stable	22 (52.4)
Unstable	20 (47.6)
Household income ($ per month)	
$0 - $1000	21 (50.0)
$1001 - $2000	16 (38.1)
> $2001	5 (11.9)
Food insecurity	
No	17 (40.5)
Yes	25 (59.5)
Sex Work as source of income	8 (19.1)
Health insurance type^a^	
Healthy SF	33 (78.6)
Medicaid and/or Medi-Cal	20 (47.6)
Medicare	25 (59.5)
Born in US	29 (69.1)

^a^ Number and percentage may exceed total sample size due to multiple responses

Table 2 describes substance use and mental health indicators. Most (85.7%) participants consumed alcohol. Of all participants, 20 used methamphetamines (47.6%), 10 used cocaine (23.8%), three used opioids (7.1%), and 10 reported polysubstance use, excluding cannabis (23.8%). Of 20 participants who used methamphetamines in the past year, 9 reported using methamphetamine daily (45.0%). Of 10 participants who used cocaine in the past year, 5 reported using cocaine daily (50.0%). Of 3 participants who used opioids, 2 reported using opioids daily (66.7%). Of 10 participants who reported polysubstance use, 3 reported using them daily (30.0%). About one-third of all participants had an AUDIT-C score of 3 or higher, indicating an alcohol use disorder (33.3%). Similarly, 28.6% of participants had a DAST score of 6 or higher, indicating a drug use disorder. Of 42 participants, 8 out of 42 had a high level of anxiety or depression (19.1%). When assessing anxiety and depression separately, 22 participants had a score indicative of anxiety (52.4%) and 17 participants had score indicative of depression (40.5%). Of all participants, about half reported high psychological distress as indicated by a Kessler score of 11 or higher (47.6%). When assessed using a cut-off score of 13, about a third of participants were classified as having serious psychological distress (33.3%).

**Table 2:** Descriptive statistics of baseline substance use and mental health among trans women living with HIV, mSN study, 2022, *N* = 42

	*N* = 42 (%)
**Substance use**	
Recent substances used^a, b^	
Alcohol	36 (85.7)
Methamphetamine	20 (47.6)
Cocaine	10 (23.8)
Opioids^c^	3 (7.1)
Polysubstance^d^	10 (23.8)
Frequency of use^a,e^	
Alcohol (*N* = 36)	
Did not use in the past year	13 (36.1)
Monthly	9 (25.0)
2–4 times a month	7 (19.4)
2–3 times a week	3 (8.3)
4 or more times a week	4 (11.1)
Methamphetamine (*N* = 20)	
Monthly	7 (35.0)
Weekly	4 (20.0)
Daily	9 (45.0)
Crack/cocaine (*N* = 10)	
Monthly	3 (30.0)
Weekly	2 (20.0)
Daily	5 (50.0)
Opioids^b^ (*N* = 3)	
Monthly	0 (0.0)
Weekly	1 (33.3)
Daily	2 (66.7)
Polysubstance^d^ use (*N* = 10)	
Monthly	3 (30.0)
Weekly	1 (10.0)
Daily	6 (60.0)
Positive screening for Alcohol Use Disorder (AUDIT-C ≥ 3)	14 (33.3)
Positive screening for a Drug Use Disorder (DAST-10 ≥ 6)	12 (28.6)
**Mental health**	
Anxiety or depression symptom level	
Not clinically significant for anxiety or depression symptom level (PHQ-4, 0–2)	7 (16.7)
Mild symptoms level (PHQ-4, 3–5)	15 (35.7)
Moderate symptoms level (PHQ-4, 6–8)	12 (28.6)
Severe symptoms level (PHQ-4, 9–12)	8 (19.1)
Positive screening for anxiety disorder (PHQ-4, anxiety subscale≥3)	22 (52.4)
Positive screening for depression disorder (PHQ-4, depression subscale ≥3)	17 (40.5)
Psychological distress	
No psychological distress (Kessler score, 0)	1 (2.4)
Low psychological distress (Kessler score, 1–5)	5 (11.9)
Moderate psychological distress (Kessler score, 6–10)	16 (38.1)
High psychological distress (Kessler score, 11–24)	20 (47.6)
Indication of serious psychological distress (Kessler score ≥ 13)	14 (33.3)

^a^Number and percentage may exceed total sample size due to multiple responses

^b^Recent substance use was defined as use in the past year

^c^Opioids included heroin, oxycodone, methadone, etc.

^d^Polysubstance included methamphetamine, cocaine, opioids, inhalants, hallucinogens, and tranquilizers

^e^Data were reported only among those who used specific substances. Denominators for each percent calculated were shown in brackets

Table 3 shows associations between substance use, mental health, and HIV viral load. Our results suggest a statistically significant association between methamphetamine use and having a detectable HIV viral load. We found that a significantly higher proportion of participants who reported using methamphetamine had an unknown or detectable HIV viral load than an undetectable viral load (71.4% vs. 35.7%, *p* = 0.049). No other significant results were detected, but descriptively 41 out of 42 participants had at least one mental health diagnosis at baseline (97.6%).

**Table 3:** Associations between HIV viral load and substance use and mental health indicators among trans women living with HIV, mSN study, 2022, *N* = 42

Substance use and mental health indicators	Unknown or detectable viral load *N* = 14 (33.3%)	Undetectable viral load *N* = 28 (66.7%)	Chi-Squared or Fisher’s Exact *P*-value
Frequent alcohol use^a^	4 (28.6)	3 (10.7)	0.197
Methamphetamine use	10 (71.4)	10 (35.7)	0.049
Polysubstance use^b^	6 (42.9)	4 (14.3)	0.059
Positive screening for alcohol use disorder	5 (35.7)	9 (32.1)	0.817
Positive screening for drug use disorder	5 (35.7)	7 (25.0)	0.469
Serious Psychological distress	5 (35.7)	9 (32.1)	0.817
Mental health diagnoses	13 (92.9)	28 (100)	0.333
Substance use diagnosis	8 (57.1)	21 (75.0)	0.298

^a^Frequent alcohol use was defined as having a drink containing alcohol 2–3 times a week or more often

^b^Polysubstance included methamphetamine, cocaine, opioids, inhalants, hallucinogens, and tranquilizers

**Fig. 1:**
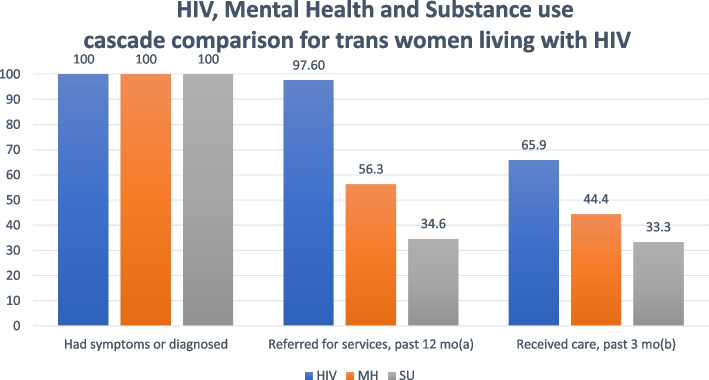
HIV, Mental Health and Substance use cascade comparison for trans women living with HIV. ^a^ Label for blue bar “referred for services, past 12 mo” is the proportion of trans women participants are currently taking HIV meds (blue). ^b^ Label for blue bar “received care, past 3 mo” is the proportion of trans women with an undetectable viral load (blue)

Figure 1 compares indicators along the HIV, mental health and alcohol/substance use care cascades. All participants living with HIV had been diagnosed (*N* = 42, 100.0%), which was an eligibility criterion for the study, and almost all had recently been referred for HIV care (*N* = 41, 97.6%). Of 41 who had been referred for HIV care, all were currently receiving HIV care (*N* = 41, 100.0%). Of 41 who ever received HIV care and were currently taking HIV medications, 27 had an undetectable HIV viral load (65.9%). Of 32 trans women who reported symptoms of psychological distress, slightly more than half were referred for mental health services in the past 12 months (56.3%). Less than half of those who were referred received mental health services (44.4%). Of 26 trans women who screened positive for a substance use disorder, less than half were referred for substance use services in the past 12 months (34.6%). Among those who were referred, about a third received substance use services in the past 3 months (33.3%).

Table 4 describes the association between demographic characteristics and behavioral factors and mental health and substance use services engagement indicators. There was a higher proportion of young trans women (30–39 years old) who screened positive for a mental health (87.5%) and drug use disorder (75.0%) than older trans women (those older than 39 years of age). There was a higher proportion of young trans women (30–39 years old) who were referred for mental health services (71.4%) than all other age groups of trans women. Only 33.3% of young trans women (30–39 years old) who screened positive for a drug use disorder was referred for substance use services. All trans women who identified as Latina screened positive for a mental health disorder. Only half were referred for mental health services (50%). All Asian/PI trans women who screened positive for a mental health disorder were referred and engaged in mental health services. Of all Asian/PI trans women who screened positive for a drug use disorder, almost all were referred for substance use services (83.3%), but only 40% were currently receiving substance use services. Almost half of trans women who identified as Black/African American screened positive for a drug use disorder (46.2%), but only 16.7% of them were referred for substance use treatment, and none were in treatment. All trans women who said sex work as their main income source screened positive for a substance use and mental health disorder, but only half were referred for mental health services (50%), and less than half were referred for substance use services (37.5%). Of 19 trans women who reported methamphetamine use and screened positive for a drug use disorder, less than half were referred for substance use services (36.8%).

**Table 4:** Bivariate analyses on factors associated with mental health and substance use treatment cascade variables, *N* = 42

	Positive screening for mental health (MH) disorder *N* = 32 (76.2%)	*P*-value	Referred for MH services, past 12 mo *N* = 18 (56.3%)	*P*-value	Received MH care, past 3 mo *N* = 8 (44.4%)	*P*-value	Positive screening for substance use (SU) disorder *N* = 26 (61.9%)	*P*-value	Referred for SU services, past 12 mo *N* = 9 (34.6%)	*P*-value	Received SU care, past 3 mo *N* = 3 (33.3%)	*P*-value
Age group^a^		0.774		0.628		0.771		0.895		0.944		0.476
30–39	7 (87.5)		5 (71.4)		2 (40.0)		6 (75.0)		2 (33.3)		1 (50.0)	
40–49	9 (81.8)		5 (55.6)		3 (60.0)		7 (63.6)		3 (42.9)		2 (66.7)	
50–59	10 (71.4)		6 (60.0)		3 (50.0)		8 (57.1)		2 (25.0)		0	
60+	6 (66.7)		2 (33.3)		0		5 (55.6)		2 (40.0)		0	
Race/ethnicity		0.060		0.236		0.037		0.114		0.025		1.000
African American/Black	7 (53.9)		4 (57.1)		1 (25.0)		6 (46.2)		1 (16.7)		0	
Latina	12 (100)		6 (50.0)		2 (33.3)		7 (58.3)		3 (42.9)		1 (33.3)	
White	4 (66.7)		2 (50.0)		0		5 (83.3)		0		0	
Asian/Pacific Islanders	5 (83.3)		5 (100)		5 (100)		6 (100)		5 (83.3)		2 (40.0)	
Other	4 (80.0)		1 (25.0)		0		2 (40.0)		0		0	
Food insecurity		0.062		0.267		1.000		0.353		0.098		0.333
No	10 (58.8)		4 (40.0)		2 (50.0)		9 (52.9)		1 (11.1)		1 (100)	
Yes	22 (88.0)		14 (63.6)		6 (42.9)		17 (68.0)		8 (47.1)		2 (25.0)	
Sex work		0.165		0.703		0.275		0.016		1.000		1.000
No	24 (70.6)		14 (58.3)		5 (35.7)		18 (52.9)		6 (33.3)		2 (33.3)	
Yes	8 (100)		4 (50.0)		3 (75.0)		8 (100)		3 (37.5)		1 (33.3)	
Methamphetamine use		0.863		0.755		0.054		< 0.001		1.000		0.500
No	17 (77.3)		10 (58.8)		2 (20.0)		7 (31.8)		2 (28.6)		0	
Yes	15 (75.0)		8 (53.3)		6 (75.0)		19 (95.0)		7 (36.8)		3 (42.9)	

*MH* mental health, *SU* substance use

^a^Row percentages are displayed when results are stratified by age, race/ethnicity, food insecurity, sex work, and methamphetamine use

## Discussion

We observed significant unmet need for substance use and mental health referrals and services among trans women living with HIV. Positive screenings for anxiety, depression, and psychological distress were prevalent and level of distress was acute among many participants. Half of trans women living with HIV had symptoms of depression and anxiety, and about one-fifth reported symptoms of severe psychological distress. Almost all trans women in our study used substances with about one-third screening positive for an alcohol and/or substance use disorder. Findings of high psychological distress are consistent with another recent large study of trans women living with HIV in Canada [25]. A quarter of our participants reported polysubstance use, which is known to be associated with ART non-adherence [26]. Trans women in our study were also living on very little income, and half were unstably housed, which may explain why we observed high psychological distress and substance use, which are known correlates of structural disadvantage among marginalized populations facing intersectional stigma and discrimination [27]. Despite the numerous barriers, most trans women in our study were highly engaged in their HIV care.

We also found that methamphetamine use was associated with having an unknown or detectable viral load. The reasons trans women may use methamphetamine are varied. A recent study found that substance use among trans women overall was associated with unmet gender-affirming healthcare needs, lack of health insurance, a history of experiencing racial violence, transphobic violence, adult housing instability, extreme poverty, and sex work [28]. Trans women may be using methamphetamine to cope with the many traumas they have experienced related to identity-based stigma and discrimination [29], and their needs for survival by facilitating the ability to do sex work [30]. Methamphetamine is a known barrier to viral suppression for trans women [27], as it is among other populations living with HIV [31]. Having a detectable viral load in our sample may be related to missed HIV care visits as has been found among San Francisco public health system primary care patients [32]. Interventions focused on services to address methamphetamine use may have important effects on overall wellness and positive HIV outcomes among trans women living with HIV.

Unmet mental health needs even among trans women who were highly engaged in their HIV care may be related to several factors. From an individual perspective, mental health stigma is a known barrier to engagement in mental health treatment [33]. Minority stress from racial and anti-trans stigma on top of HIV stigma may exacerbate psychological distress while also reducing help-seeking for mental health support [34]. From a systems perspective, a key component to ensure success of integrated and culturally tailored substance use and mental health treatment is screening and referral [35]. We found that referrals for trans women with indicators of mental distress were low, suggesting that screening protocols may not be in place for behavioral health needs. From a social determinants perspective, existing mental health referrals in the public health system are predominantly focused on in-person counseling. Trans women with competing needs for housing, safety, food, and other instrumental needs may not be able to attend or commit to regular therapy within the confines of current delivery models [36, 37]. Discrimination is also a deterrent to accessing health care [38, 39]. In a recent study with 629 trans women living in San Francisco, the majority of participants (93%) reported experiencing anti-trans discrimination and 88.1% reported transphobic violence [28]. Another study found that almost a quarter of trans women in San Francisco reported discrimination in accessing health and medical services [40].

Similarly, only about one-third of participants with indications of a substance use disorder was referred for substance use services, and of those, only about one-third received services. Healthcare provider referrals to substance use treatment warrant further investigation. A large national study using data from treatment facilities found that healthcare provider referrals comprised only about 10% of all referrals for substance use treatment, and referrals from healthcare providers were associated with the least success in substance use treatment [41, 42]. Authors found that healthcare providers preferred short-term detoxification over outpatient treatment, which may be why treatment success with healthcare provider referrals was low [41]. The lack of referral and engagement in substance use services may also be related to the high methamphetamine use among trans women in our study, for which there is limited evidence of efficacious treatment options. Different from opioids, there is currently no established pharmacotherapy for the treatment of amphetamine or methamphetamine [43]. There are known cognitive behavioral therapies (CBT) available for the treatment of methamphetamine, but the evidence is not conclusive as to the impact of CBT on treating methamphetamine use [44]. Also, trans women who were suffering from the effects of a substance use disorder compounded with other social determinants of health barriers may not have the resources, support, or ability to engage in substance use treatment [7]. The overall lack of trans-specific substance use interventions may also be a barrier. A systematic review of tailored substance use interventions for trans people conducted in 2017 only found two studies upon which to report [45].

We also observed racial/ethnic health inequities in terms of referrals provided to participants for substance use and mental health services. Very few Black/African American trans women living with HIV who screened positive for a substance use disorder were referred for substance use treatment, and few Latinas living with HIV who had mental health symptoms were referred for mental health services. This is consistent with studies of populations who use substances finding that having a racial/ethnic minority identity is associated with a lower likelihood of being referred to substance use treatment [46]. Research also finds inequitable referrals for mental health among Latinos. One study found that Spanish-speaking Latinos were 72% less likely to receive mental health referrals from primary care providers with patients’ negative perceptions about medications, patient noncompliance, and a shortage of bilingual behavioral health providers within the clinic explaining why [47]. Trans women who are undocumented may be the least likely to receive referrals and access to mental health care. One study from California found that access to mental health services was worse for undocumented Latinos compared to US-born Latinos while a need for mental health referrals was higher [48].

Despite the importance of our results, we acknowledge data are from an intervention study, which creates inherent biases related to the eligibility criteria and limiting the generalizability of our results. Our eligibility criteria that trans women must be connected to the public health care system may have biased the sample, however, recent data shows that 92.5% of trans women in San Francisco reported having health insurance, with only 11.4% reporting private health insurance [3]. We also recognize that our small sample size may not be sufficient to detect significance in our results. Despite these limitations, we did identify a need for interventions to improve behavioral health engagement among trans women living with HIV with a focus on mitigating health inequities within the population. Integration of outpatient substance use services and low threshold mental health services into HIV care may improve engagement [35, 49]. Promising examples are emerging from the integration of opioid agonist treatment (OAT) services that are embedded within HIV care. One study in Ukraine found much better HIV care outcomes among people living with HIV who had integrated OAT and HIV care [50], and another study found improvements in higher order HIV care indicators once on OAT [51]. Novel low threshold interventions to meet the mental health needs of trans women living with HIV are also needed.

Only half of those who did sex work who had mental health symptoms were referred to mental health services. Routine screening for mental health and substance use disorders may address the unmet mental health and substance use needs found among trans women. However, public health and community clinics may not be adequately resourced to do so. Staffing shortages and a large patient load with complex needs may be a reason for fewer referrals to mental health and substance use services. Shifting tasks such as screening to a trained clinic staff person with the help of an electronic screener or an app-based mental health or substance use screener may enable referrals. Other modalities of screening such as a self-administered electronic screener for mental health and substance use have been found acceptable for patients in HIV primary care [52].

Many trans women who are competing with meeting basic needs may not be able to attend regular counseling sessions for mental health. And services only offered during regular business hours may make engagement impossible even when referred. There is a need for interventions to change the system of delivery for people who face structural barriers so that those most in need are able to be engaged. There may also be a need to offer alternatives to counseling that are more amenable to trans women’s barriers like income and safety to improve engagement in services. With careful consideration of the needs of trans women, healthcare systems have much to gain to improve the well-being and healthcare outcomes of trans women living with HIV.

## Data Availability

The data that support these findings of this study is available from the authors upon reasonable request.
